# Population-based rates, risk factors and consequences of preterm births in South-Asia and sub-Saharan Africa: A multi-country prospective cohort study

**DOI:** 10.7189/jogh.12.04011

**Published:** 2022-02-19

**Authors:** The Alliance for Maternal and Newborn Health Improvement (AMANHI) GA Study Group, Fahad Aftab, Parvez Ahmed, Salahuddin Ahmed, Corneille Bashagaluke Akonkwa, Said Mohammed Ali, Rajiv Bahl, Bowen Banda, Abdullah H Baqui, Nazma Begum, Godfrey Biemba, Sayan Das, Saikat Deb, Usha Dhingra, Arup Dutta, Karen Edmond, Davidson H Hamer, Julie Herlihy, Lisa Hurt, Fyezah Jehan, Mohamed Hamad Juma, Monica Lulu Kapasa, Muhammad Karim, Farzana Kausar, Farah Khalid, Betty R Kirkwood, Anne CC Lee, Alexander Manu, Usma Mehmood, Dipak Mitra, Fern Mweene, Naila Nadeem, Muhammad Imran Nisar, Rina Paul, Mahmoodur Rahman, Sayedur Rahman, Muhammad Sajid, Sunil Sazawal, Katherine E Semrau, Shahira Shahid, Caitlin Shannon, CARE USA, New York, New York, Marina Straszak-Suri, Atifa Suleiman, Mohammad J Uddin, Jayson Wilbur, Blair Wylie, Sachiyo Yoshida

## Abstract

**Background:**

Preterm birth is the leading cause of neonatal deaths in low middle-income countries (LMICs), yet there exists a paucity of high-quality data from these countries. Most modelling estimates are based on studies using inaccurate methods of gestational age assessment. We aimed to fill this gap by measuring the population-based burden of preterm birth using early ultrasound dating in five countries in South-Asian and sub-Saharan Africa.

**Methods:**

We identified women early in pregnancy (<20 weeks based on last menstrual period) by home visits every 2-3 months (except in Zambia where they were identified at antenatal care clinics) in 5 research sites in South-Asia and sub-Saharan Africa between July 2012 and September 2016. Trained sonographers performed an ultrasound scan for gestational age dating. Women were enrolled if they were 8-19 weeks pregnant on ultrasound. Women <8 weeks were rescheduled for repeat scans after 4 weeks, and identified women were followed through pregnancy until 6 weeks postpartum. Site-specific rates and proportions were calculated and a logistic regression model was used to predict the risk factors of preterm birth.

**Results:**

Preterm birth rates ranged from 3.2% in Ghana to 15.7% in Pakistan. About 46% of all neonatal deaths occurred among preterm infants, 49% in South Asia and 40% in sub-Saharan Africa. Fourteen percent of all preterm infants died during the neonatal period. The mortality was 37.6% for early preterm babies (<34 weeks), 5.9% for late preterm babies (34 to <37 weeks), and 1.7% for term babies (37 to <42 weeks). Factors associated lower gestation at birth included South-Asian region (adjusted mean difference (Adj MD) = -6.2 days, 95% confidence interval (CI) = -5.5, -6.9), maternal morbidities (Adj MD = -3.4 days, 95% CI = -4.6, -2.2), multiple pregnancies (Adj MD = -17.8 days, 95% CI = -19.9,-15.8), adolescent pregnancy (Adj MD = –2.7 days, 95% CI = -3.7, -1.6) and lowest wealth quintile (Adj MD = -1.3 days, 95% CI = -2.4, -0.3).

**Conclusions:**

Preterm birth rates are higher in South Asia than in sub-Saharan Africa and contribute to 49% and 40% of all neonatal deaths in the two regions, respectively. Adolescent pregnancy and maternal morbidities are modifiable risk factors associated with preterm birth.

Preterm birth is a leading cause of death in children under five years of age worldwide. Approximately 14.8 million babies were born preterm in 2014, accounting for 10.6% of all live births globally [1]. In addition, about one million babies die due to direct complications of being born preterm, ie, before completing 37 weeks of gestation [2,3]. Globally, the preterm birth rate increased from 9.8% (8.3-10.9) in 2000 to 10.6% (9.0-12.0) in 2014. South Asia and sub-Saharan Africa alone contribute to 81.1% of these preterm births, with countries like India, China, Nigeria, Bangladesh, Indonesia and Pakistan having 7.0 million (47.7%) of preterm births globally in 2014 [1,2].

The cut off for preterm birth defined above is, to an extent, arbitrary. Recent studies show that neonatal outcomes vary within the term group, with greater risks of adverse outcomes associated with early-term neonates than those born at 40 weeks of gestational age [4]. In most low-income settings, where facilities for obstetric ultrasound are not available, the gestational age of the fetus or the newborn is calculated by counting weeks passed since the first day of the last menstrual period (LMP). This is inherently dependent on pregnant women’s recall and can be highly inaccurate at times [5,6]. The “gold standard” for measuring gestational age is first-trimester obstetric ultrasound [7-9]. Other methods used to estimate gestational age include symphysis-fundal height, birth weight, and clinical assessment of newborn after birth [10-12] having varying levels of inaccuracy, eg, using birthweight vastly overestimate the number of preterm births, particularly in South-Asian countries where many babies are born small for gestational age (SGA) [13,14].

The preterm birth syndrome involves a complex interplay between various sociodemographic, environmental, and biological factors. Some of the factors identified in the literature include black sub-Saharan African ancestry, family history, prior history of preterm births and an interpregnancy interval of fewer than six months [2,15]. Other maternal factors involved are young age, under and over-nutrition, infections, bleeding during pregnancy, and history of substance abuse. In addition, multiple pregnancies carry 10 times the risk of preterm births compared to singleton pregnancies [15,16].

While several studies have showcased global estimates and levels of preterm births, there is a general agreement about the gaps in existing data, particularly from South-Asian and sub-Saharan Africa. One important reason for this disproportionate reporting of data are absent or incomplete registries [17]. Even though WHO guidelines recommend antenatal ultrasounds for all pregnant women, this is rarely practiced for various reasons [18]. There is also misclassification of live births, stillbirths, and deaths which affect reporting of preterm births, including various phenotypes such as spontaneous or provider-initiated in these regions [1]. In the given scenario, it is imperative to generate accurate data on the true burden of preterm births and its associated risk factors. This will help us better understand preterm birth and develop effective primary and secondary interventions to decrease the associated morbidity and mortality.

## METHODS

The Alliance for Maternal and Newborn Health Improvement (AMANHI) gestational age group conducted a multi-country, population-based prospective cohort study to evaluate the diagnostic accuracy of simple methods for gestational age assessment (including reported LMP, physical, neuromuscular, feeding assessments, and anthropometry). These methods were compared with gestational age calculated from early pregnancy ultrasound scans. The study protocol was previously published [19].

We conducted a study in five community sites in South-Asia (Sylhet, Bangladesh, and Karachi, Pakistan), sub-Saharan Africa (Kintampo, Ghana; Pemba, Tanzania; and Southern Province, Zambia) between July 2012 and September 2016. The detailed methods for enrollment and follow up are described elsewhere [20]. Briefly, in 4 out of 5 sites, pregnant women were identified at home using 2 to 3 monthly household surveillance, offered a pregnancy test, and a gold standard ultrasound scan for gestational age dating. Sonographers at all sites received centralized training coordinated by the WHO MCA department. Women were enrolled if they were between 8-19 weeks pregnant on ultrasound. Women <8 weeks were rescheduled for repeat scans after 4 weeks and enrolled at ≥8 weeks gestation. At 20 weeks or more, pregnant women were counselled to continue routine antenatal clinic attendance but were not enrolled on the AMANHI gestational age study.

We measured only the crown-rump length (CRL) if the pregnancy was less than 14 weeks (CRL<95mm), both biparietal diameter (BPD), and femur length (FL) if more than 14 weeks, and all three if within the 14th week. Thus, we conducted three measurements for each biometric parameter. We promptly referred women with major abnormalities or intrauterine fetal deaths to health facilities for appropriate management. The preterm birth rate was defined as all live births before 37 completed weeks of gestation divided by the total number of live births. Early to moderate preterm birth rate was defined as live births before 34 completed weeks of gestation, divided by the total live births. Small for gestational age was defined as newborns who were less than 10th centile for their gestational age using INTERGROWTH criterion [20].

We established a birth notification system to capture birth outcomes as close to birth as possible, which included active surveillance, reporting by family members and key informants. All live births were then followed till 28 days of life to ascertain vital status. In case mortality was recorded, we conducted a detailed verbal autopsy interview after a culturally appropriate mourning period.

Data were collected on paper and entered electronically at all sites except Zambia, where filled forms were scanned using TeleForms Software (Hewlett Packard, Sunnyvale, CA). Sites shared data to WHO quarterly for quality assurance and data monitoring. Any inconsistencies were flagged and resolved in consultation with data managers and investigators from the sites.

Logistic regressions were performed to examine the association of gestational age and neonatal mortality and identify risk factors of preterm birth. A multivariate model was adjusted for variables with a *P* value of ≤0.10 at the univariate model and the site. In the analysis on the risk factor of preterm birth, we used different hierarchical models to obtain adjusted odds ratios (ORs) [21]. We calculated the mean difference in gestation for different factors associated with the duration of gestation among women with the first postnatal visit. This analysis performed the imputation of missing values by mean imputation for height and the frequent category for all other covariates. The site was put in as a factor variable. Site interactions were tested to identify variables whose effects might vary across sites. Site-specific gestational age distributions were plotted using both LMP and gold standard ultrasound. A Bland Altman plot was created for the difference in gestational age estimates between the gold standard and the LMP (Ultrasound based gestational age-LMP). All analysis was carried out using Stata version 16 (Stata Corp, College Station, TX, USA).

### Ethical approval

Ethical approval was obtained from WHO Ethical Review Committee (ERC) and all site ERC’s. Fieldworkers obtained consent from pregnant women in their local or preferred language.

## RESULTS

**Figure 1:**
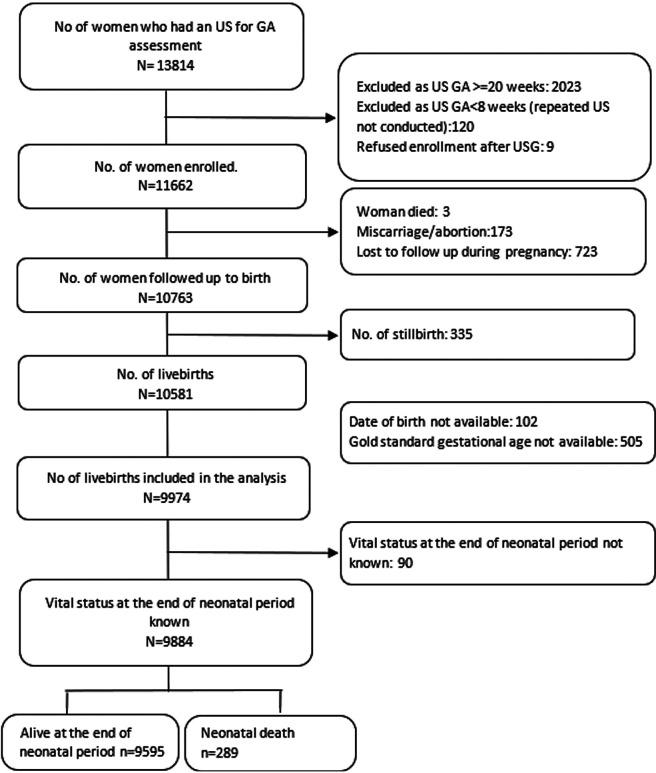
Flowchart of study participants (all sites combined).

Of 13 814 women who had an ultrasound for gestational age assessment, 11 662 (84%) were enrolled. Of them, 10 763 (92%) women were followed up till birth. There were 10 581 live births registered in the study, of which 9974 were included in the analysis (Figure 1). Vital status at the end of the neonatal period was known for 9884 live births. Baseline characteristics of enrolled women with the known birth outcome are described for each site (**Table 1**). Most pregnant women were in the age group 20-34 years old. The level of their education varied between the sites, with more than half of women in Pakistan (59.2%) having no formal education, to the majority having some level of education (86%) in Zambia. The highest previous child death rate was reported in Ghana (21.7%), the highest previous stillbirth rate in Bangladesh (13.1%) and the highest previous preterm rate was reported by mothers in Zambia (3.7%). South-Asian women were on average shorter in height as compared to their sub-Saharan African counterparts. Antepartum haemorrhage ranged from a low of 0.3% of women in Bangladesh to a high of 3.6% in Pakistan. Similarly, later antepartum infection (fever before or during delivery) was highest in the Pakistan site (12.4%). Pre-eclampsia or eclampsia was present in less than 1% of women in all sites except in Tanzania (4.7%). The rate of multiple gestations was highest in Ghana and Tanzania (3.9%). Majority of women in sub-Saharan African sites delivered at a health facility in the presence of a skilled birth attendant, while only 49.0% of births in Bangladesh and 65.3% in Pakistan occurred at a health care facility. The proportion of women having normal vaginal deliveries ranged from (85.4%-96.7%). Of the 9173 term births (≥37 weeks), 2.0% resulted in stillbirth, and of 1114 preterm birth (<37 weeks), 11.9% resulted in stillbirth (data not shown).

**Table 1:** Baseline characteristics of enrolled pregnant women (n = 10 763)

	South Asian sites		sub-Saharan African sites		
	**Bangladesh (N = 2982)**	**Pakistan (N = 2608)**	**Ghana (N = 976)**	**Tanzania (N = 2427)**	**Zambia (N = 981)**
**Characteristics of woman and family:**					
Pregnant woman's age, n (%)	2331	2171	970	2420	962
15-19	458 (19.7)	163 (7.5)	85 (8.8)	187 (7.7)	276 (28.7)
20-34	1799 (77.2)	1788 (82.4)	735 (75.8)	1764 (72.9)	579 (60.2)
35+	74 (3.2)	220 (10.1)	150 (15.5)	469 (19.4)	107 (11.1)
Woman's education, n (%)	2891	2171	970	2420	956
None	249 (8.6)	1285 (59.2)	280 (28.9)	310 (12.8)	18 (1.9)
1-6 y	1207 (41.8)	358 (16.5)	597 (61.6)	832 (34.4)	97 (10.2)
7-12 y	1415 (49)	510 (23.5)	77 (7.9)	1246 (51.5)	822 (86)
13 + years	20 (0.7)	18 (0.8)	16 (1.7)	32 (1.3)	19 (2)
Previous child death n (%)	2005	1731	801	1895	674
≥1 child death	393 (19.6)	154 (8.9)	174 (21.7)	299 (15.8)	90 (13.4)
Previous stillbirth, n (%)	2005	1905	801	1967	674
≥1 stillbirth	262 (13.1)	160 (8.4)	74 (9.2)	176 (9)	16 (2.4)
Previous preterm birth, n (%)	2001	1892	801	1895	672
Yes	30 (1.5)	48 (2.5)	13 (1.6)	34 (1.8)	25 (3.7)
Maternal height, n (%)	2156	953	798	2121	868
Mean (SD)	150.1 (5.1)	155.1 (5.9)	158.0 (6.9)	155.2 (6.4)	160.2 (7.6)
**Household characteristics:**					
Clean cooking fuel, n (%)	2914	2171	969	2420	954
Yes	34 (1.2)	1953 (90)	100 (10.3)	350 (14.5)	66 (6.9)
Improved latrine facility*, n (%)	2977	2171	969	2420	961
Yes	2876 (96.6)	2085 (96)	768 (79.3)	1781 (73.6)	889 (92.5)
Wealth quintile, n (%)	2971	2171	970	2420	622
Poorest	560 (18.9)	429 (19.8)	196 (20.2)	488 (20.2)	131 (21.1)
Poorer	597 (21.0)	435 (20.0)	207 (21.3)	512 (21.2)	103 (16.6)
Middle	604 (20.3)	434 (20.0)	188 (19.4)	485 (20.0)	120 (19.3)
Richer	616 (20.7)	428 (19.7)	188 (19.4)	461 (19.1)	124 (19.9)
Richest	594 (20.0)	445 (20.5)	191 (19.7)	474 (19.6)	144 (23.2)
Piped drinking water access, n (%)	2976	2171	970	2420	956
Yes	35 (1.2)	971 (44.7)	514 (53.0)	2245 (92.8)	255 (26.7)
**Morbidity during the current pregnancy:**					
Antepartum hemorrhage, n(%)	2303	1718	974	2406	968
Yes	9 (0.3)	61 (3.6)	26 (2.7)	26 (1.1)	7 (0.7)
Pre-eclampsia or eclampsia, n(%)	2301	1712	974	2406	968
Yes	15 (0.7)	11 (0.8)	8 (0.8)	112 (4.7)	0 (0)
Fever before or during delivery, n (%)	2139	1363	969	2326	566
Yes	33 (1.5)	169 (12.4)	57 (5.9)	12 (0.5)	0 (0)
**Birth characteristics:**					
Multiple birth, n (%)	2983	2608	976	2427	981
Yes	24 (0.8)	69 (2.7)	38 (3.9)	95 (3.9)	20 (2)
Childbirth in a health facility, n (%)	2845	2608	973	2329	828
Yes	1395 (49)	1699 (65.3)	784 (80.6)	2324 (99.8)	710 (85.8)
Skilled birth attendant, n (%)	2844	2604	973	2329	807
Yes	1355 (47.6)	1816 (69.7)	770 (79.1)	1716 (73.7)	649 (80.4)
Type of delivery, n (%)	2845	2,508	973	2329	858
Normal vaginal delivery	2449 (86.1)	2141 (85.4)	848 (87.2)	2241 (96.2)	830 (96.7)
Assisted vaginal delivery	48 (1.7)	47 (1.9)	8 (0.8)	8 (0.3)	3 (0.4)
C-section	348 (12.2)	320 (12.8)	117 (12)	80 (3.4)	25 (2.9)

CI – confidence interval, SD – standard deviation

*Flush/pour flush toilet.

Among the 9974 live births across five sites, preterm birth rates ranged from 3.2% in Ghana to 15.7% in Pakistan (**Table 2**). More than one in four infants were born with low birth weight (<2500 g) in South-Asian sites (26.6% in Bangladesh, 25.2% in Pakistan). Similarly, infants who were small for gestational age were more in South-Asian sites (42.7% in Bangladesh and 35.5% in Pakistan) than in sub-Saharan African sites (10.2% in Tanzania and 19.2% in Zambia) except in Ghana, showing a similar rate as for Pakistan (34.3%).

**Table 2:** Gestation and size at the time of birth for live-born infants (n = 9974)

	South Asian sites		sub-Saharan African sites		
	**Bangladesh (N = 2982)**	**Pakistan (N = 2608)**	**Ghana (N = 976)**	**Tanzania (N = 2427)**	**Zambia (N = 981)**
Gestational age at birth*					
Mean (SD)	38.9 (2.1)	38.6 (2.1)	39.9 (1.6)	39.5 (1.7)	39.8 (2.8)
Median (IQR)	39.3 (38.1, 40.1)	38.9 (37.7, 39.9)	40 (39.1, 40.9)	39.6 (38.7, 40.4)	39.9 (38.7, 40.9)
Preterm birth* (<37 wks)	11.7% (10.6%, 12.9%)	15.7% (14.3%, 17.1%)	3.2% (2.3%, 4.6%)	4.9% (4.1%, 5.8%)	7.4% (5.9%, 9.3%)
Gestational age at birth categories (%)*	2982	2608	976	2427	981
Early preterm (<34 wk)	99 (3.3%)	79 (3%)	5 (0.5%)	31 (1.3%)	30 (3.1%)
Late Preterm (34 to <37 wk)	250 (8.4%)	330 (12.7%)	27 (2.8%)	88 (3.6%)	43 (4.4%)
Term (37 to <42 wk)	2560 (85.2%)	2172 (83.3%)	887 (90.9%)	2220 (91.5%)	793 (80.8%)
Post term (≥42 wk)	74 (2.5%)	27 (1.0%)	57 (5.8%)	88 (3.6%)	115 (11.7%)
**Mean birth weight in grams†**	2752 (488)	2764 (492)	2940 (456)	3283 (521)	3107 (511)
**Low birth weight (<2500g) (%, 95% CI)**	26.6% (24.9%, 28.3%)	25.2% (23.5%, 27.0%)	13.2% (11.1%, 15.5%)	6.4% (5.4%, 7.5%)	7.9% (6.2%, 10.0%)
**Birth weight categories in grams, n(%)**	2590	2415	970	2320	787
<1500g	14 (0.5%)	18 (0.8%)	4 (0.4%)	4 (0.2%)	2 (0.3%)
1500-1999g	82 (3.2%)	108 (4.5%)	12 (1.2%)	29 (1.3%)	8 (1.0%)
2000-2499g	592 (22.9%)	482 (20.0%)	112 (11.6%)	115 (5.0%)	52 (6.6%)
2500-2999g	1142 (44.1%)	1099 (45.5%)	361 (37.2%)	472 (20.3%)	220 (28.0%)
≥3000g	760 (29.3%)	708 (29.3%)	481 (49.6%)	1700 (73.3%)	505 (64.2%)
**Small for gestational age‡, % (95% CI)**	42.7% (40.8%, 44.7%)	35.5% (33.6%, 37.4%)	34.3% (31.4%, 37.4%)	10.2% (9.0%, 11.5%)	19.2% (16.5%, 22.2%)

CI – confidence interval

*Gestational age is based on ultrasound (gold standard).

†Birth weight is missing for 392 (13.2%) in Bangladesh, 193 (7.4%) in Pakistan, 6 (0.6%) in Ghana, 107 (4.4%) in Tanzania and 194 (19.8%) in Zambia.

‡Size for gestational age is missing for 441 (14.8%) in Bangladesh, 203 (7.8%) in Pakistan, 23 (2.4%) in Ghana, 125 (5.2%) in Tanzania and 236 (24.1%) in Zambia.

**Figure 2:**
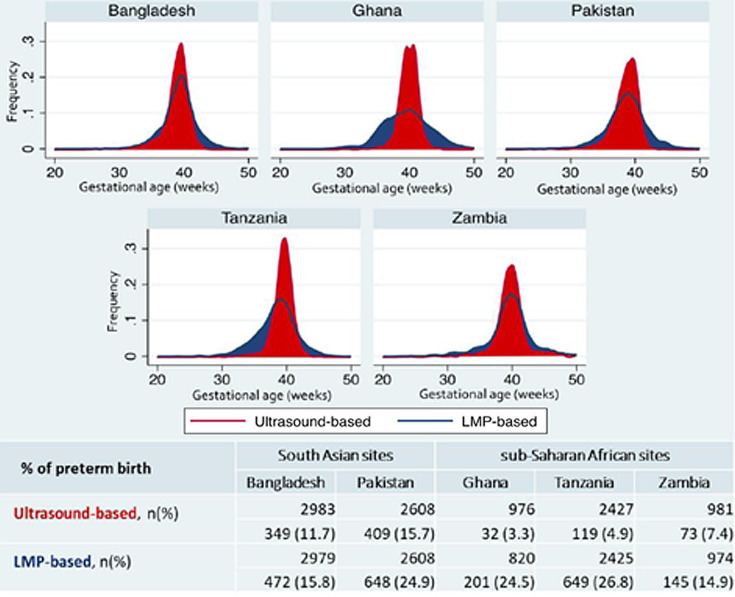
Frequency of gestational age in weeks by gestational age assessment method and site.

Estimates for preterm birth rate were much higher than ultrasound-based gestational age when LMP was used for estimation (Figure 2). The difference in the burden of preterm birth by two measures was more pronounced in sub-Saharan African sites. However, the LMP based preterm birth was similar in the two regions.

Comparison of gestational age at the time of ultrasound by two measurements (ultrasound vs LMP) showed that the 95% limits of agreement ranged from -35.2 to 39.1 days (Mean bias = 1.95 days; standard deviation (SD) = 18.95) (Figure S1 in the **Online Supplementary Document**). Thus, LMP tends to overestimate gestational age at earlier gestation and underestimate at a later gestation.

Of the 9884 live births, 289 (2.9%) resulted in neonatal deaths (**Table 3**). The neonatal mortality rate was slightly higher in South-Asian sites than in sub-Saharan African sites (3.6% in South-Asian sites and 2.0% in sub-Saharan African sites). Infants born preterm accounts for approximately 46% of neonatal deaths (49% in South-Asia and 40% in sub-Saharan African sites). About 16% of preterm infants in sub-Saharan African sites and 13% in South-Asian sites died during the neonatal period.

**Table 3:** Neonatal deaths by gestational age at birth, overall and by region

Overall					
Live births whose vital status at the end of neonatal period is known	9884				
Neonatal deaths	289 (29/1000 LB)				
Proportion of preterm deaths among all neonatal deaths	46.4%				
Proportion of preterm infants who died during neonatal period	13.8%				
Gestational age* in completed week (gestational age available n = 9884)	**Neonatal deaths**	**OR (95% CI)**	**Adjusted OR**† **(95% CI)**		***P-*value**
<32 weeks (n = 98)	61 (62.2%)	98.4 (63.3, 152.9)	108.2 (52.1, 224.1)		
32 to <34 weeks (n = 144)	30 (20.8%)	15.7 (10.2, 24.3)	13.7 (6.9, 27.2)		
34 to <37 weeks (n = 732)	43 (5.9%)	3.7 (2.6, 5.3)	3.6 (2.1, 6.3)		*P* < 0.001
37 to <42 weeks (n = 8556)	141 (1.7%)	1.0 (reference)	1.0 (reference)		
≥42 weeks (n = 354)	14 (4.0%)	2.5 (1.4, 4.3)	1.9 (0.67, 5.4)		
**Asia**					
Live births whose vital status at the end of neonatal period is known	5563				
Neonatal deaths	201 (36.1/1000 LB)				
Proportion of preterm deaths among all neonatal deaths	49.3%				
Proportion of preterm infants who died during neonatal period	13.2%				
gestational age* in week (gestational age available n = 5563)	**Neonatal deaths**	**OR (95% CI)**		**Adjusted OR**†**(95% CI)**	***P*-value**
<32 weeks (n = 69)	45 (65.2%)	93.1 (54.4, 159, 2)		162.2 (61.5, 427.7)	
32 to <34 weeks (n = 109)	18 (16.5%)	9.8 (5.7, 17.0)		9.8 (4.1, 23.5)	
34 to <37 weeks (n = 574)	36 (6.3%)	3.3 (2.2, 4.9)		4.0 (2.1, 7.6)	*P* < 0.001
37 to <42 weeks (n = 4712)	93 (2.0%)	1.0 (reference)		1.0 (reference)	
≥42 weeks (n = 99)	9 (9.1%)	4.7 (2.4, 10.2)		3.0 (0.7, 13.2)	
**Africa**					
Live births whose vital status at the end of the neonatal period is known	4321				
Neonatal deaths	88 (20.4/1000 LB)				
Proportion of preterm deaths among all neonatal deaths	39.8%				
Proportion of preterm infants who died during neonatal period	15.8%				
Gestational age* in week (gestational age available n = 4321)	**Neonatal deaths**	**OR (95% CI)**		**Adjusted OR**† **(95% CI)**	***P-*value**
<32 weeks (n = 29)	16 (55.2%)	97.3 (44.4, 213.5)		65.7 (17.8,242.2)	
32 to <34 weeks (n = 35)	12 (34.3%)	41.3 (19.4, 87.7)		39.5 (10.6, 146.0)	
34 to <37 weeks (n = 158)	7 (4.4%)	3.7 (1.6, 8.2)		2.7 (0.8, 9.3)	*P* < 0.001
37 to <42 weeks (n = 3844)	48 (1.3%)	1.0 (reference)		1.0 (reference)	
≥42 weeks (n = 255)	5 (2.0%)	1.6 (0.6, 4.0)		1.3 (0.3, 5.4)	

OR – odds ratio, CI – confidence interval

*Gestational age is based on ultrasound.

†OR was adjusted for site, mother's age, mother's education, wealth quintile and previous obstetric history, including previous stillbirth and previous preterm birth, morbidity during pregnancy (antepartum haemorrhage, pre-eclampsia and eclampsia, antepartum fever) and multiple gestations.

**Figure 3:**
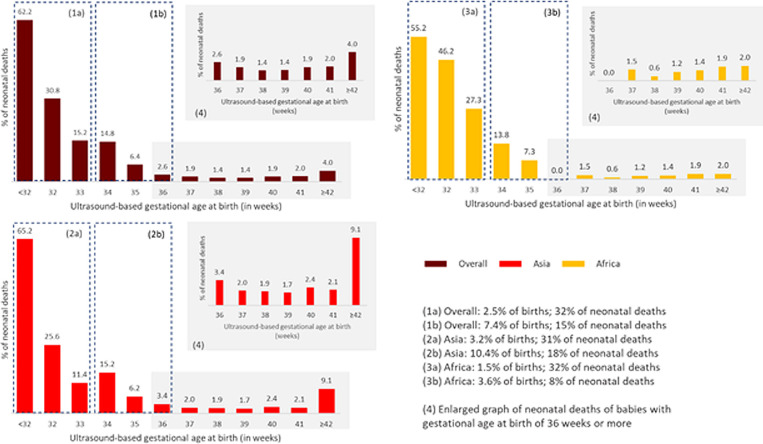
Risk of neonatal death by gestational age, overall and by region.

The risk of neonatal deaths in very preterm infants (<32 weeks) was 62.2% (adjusted odds ratio (aOR) = 108.2, 95% confidence interval (CI) = 52.1, 224.1; South-Asia aOR = 162.0, 95% CI = 61.5, 427.7; sub-Saharan Africa aOR = 65.7, 95% CI = 17.8, 242.2). Risk of neonatal mortality in moderate preterm infants (32 to <34 weeks) was 20.8% (aOR = 13.7, 95% CI = 6.9, 27.2; South-Asia aOR = 9,8, 95% CI = 4.1, 23.5; *P* < 0.001; sub-Saharan Africa aOR = 39.5, 95% CI = 10.6, 146.0; *P* < 0.001) (**Table 3**; **Figure 3**).

The mean duration of gestation was 274.6 days (SD = 13.8), and the duration was shorter by 6.2 days in South-Asian sites than in sub-Saharan African sites (adjusted mean difference (aMD) = 6.2 days, 95% CI = 5.5, 6.9) (**Table 4**). Mean gestational age at birth was 18.1 days shorter in case of multiple pregnancies (aMD = -17.8, 95% CI = -19.9, -15.8), adolescent pregnancy (aMD = -2.7 days, 95% CI = -3.7, -1.6) and low socioeconomic status (poorest wealth quintile) (aMD = -1.3 days, 95% CI = -2.4, -0.3).

**Table 4:** Explanatory factor associated with duration of gestation, among women with the first postnatal visit (n = 7031)

Ultrasound-based gestational age	Overall (n = 7031)		
Mean (SD)- in days*	274.6 (13.8)		
**Explanatory factors**	**Effect size**		
	n/N (%)	MD (95% CI)†	AdjMD (95% CI)‡
Region:			
Asia (ref)	3162/7031 (45.0)	1	1
Africa	3869/7031 (55.0)	6.2 (5.5; 6.8)	6.2 (5.5; 6.9)
Cesarean section (ref: vaginal birth)	610/7017 (8.7)	-0.6 (-1.8;0.5)	-0.4 (-1.5;0.8)
Obstetric history:			
Primipara	1537/6910 (22.2)	-0.4 (-1.1; 0.4)	0.6 (-0.3;1.4)
Multipara with previous stillbirth	469/6910 (6.8)	-1.7 (-3.0; -0.4)	-1.2 (-2.5;0.0)
Multipara with previous preterm birth or with previous stillbirth and previous preterm birth	99/6910 (1.4)	0.9 (-3.6;1.8)	-0.8 (-3.4;1.9)
Multipara with no previous stillbirth or preterm birth (ref)	4805/6910 (69.5)	1	1
Morbidity during pregnancy (ref: no)§	509/7009 (7.3)	-3.8 (-5.0; -2.6)	-3.4 (-4.6; -2.2)
Multiple pregnancy (ref: singleton pregnancy)	163/6868 (2.3)	-18.1 (-20.2; -16.1)	-17.8 (-19.9; -15.8)
Adolescent (15-19y) (ref: >19 y)	900/7015 (12.8)	-2.0 (-2.9; -1.0)	-2.7 (-3.7; -1.6)
No education (ref: some education)	1508/6994 (21.6)	-0.3 (-1.1;0.5)	0.1 (-0.7;0.9)
Maternal height (per cm)‖	154.5 (7.1)	0.1 (0.0;0.1)	0.1 (0.0;0.1)
Wealth quintile:			
Poorest	1412/7010 (20.1)	-1.4 (-2.4; -0.4)	-1.3 (-2.4; -0.3)
Poor	1416/7010 (20.2)	-1.2 (-2.2; -0.2)	-0.9 (-1.9;0.1)
Middle	1416/7010 (20.2)	-0.7 (-1.7;0.3)	-0.6 (-1.6;0.4)
Rich	1363/7010 (19.4)	-0.4 (-1.4;0.6)	-0.3 (-1.3;0.7)
Richest (ref)	1403/7010 (20.0)	1	1

MD – mean difference

*Gestational age at birth in days by site [mean(sd)]: Asia: Bangladesh 271.9 (14.5), Pakistan 270.2 (14.2); Africa: Ghana 279.5 (10.9), Tanzania 276.3 (11.8), Zambia 278.1 (17.6).

†Adjusted for region.

‡Adjusted for type of delivery, maternal age, maternal education, wealth quintile, multiple births, obstetric history, and any maternal morbidity (eclampsia or pre-eclampsia, antepartum hemorrhage, fever.

§Morbidity during pregnancy includes pre-eclampsia or eclampsia or fever before delivery or antepartum hemorrhage.

‖Mean.

Multivariate analyses examining the risk factor of preterm birth (all preterm and spontaneous preterm) showed consistent results (Tables S1 and S2 in the **Online Supplementary Document**). The factors associated with preterm birth included: younger maternal age group of 15-19 years (aOR = 1.27, 95% CI = 1.03, 1.58), low socioeconomic status (aOR = 1.36 95%, 95% CI = 1.05, 1.77), history of the previous stillbirth (aOR 1.84, 95% CI = 1.42, 2.37) and previous preterm birth (AdjOR 1.96, 95% CI = 1.18, 3.26). Other pregnancy-related risk factors included pre-eclampsia /eclampsia (aOR = 2.74, 95% CI = 1.56, 4.84), fever before or during delivery (aOR = 1.76, 95% CI = 1.18, 2.62), and multiple gestations (aOR = 14.3, 95% CI = 9.92, 20.52).

## DISCUSSION

This was a large population-based study reporting preterm birth rates across South-Asian and sub-Saharan African sites using gestational age based on ultrasound scan. In our study, we found that preterm birth rates were much higher in South-Asian sites than in sub-Saharan African sites, from 15.7% in Pakistan to 3.2% in Ghana.

We found higher proportions of LBW and SGA babies in South-Asian sites. This could be due to the poor nutritional status of these women. Nearly a quarter of women were underweight in South-Asian sites while only 5.8% in sub-Saharan African sites in AMANHI biorepository cohort- unpublished data. Micronutrient and protein-energy supplementation provided to pregnant mothers seem to significantly reduce SGA births and low birth weights [22,23]. Unfortunately, we did not collect information on maternal nutritional status in this study. Maternal height can be another independent risk factor. A previously published meta-analysis showed that a large proportion of preterm birth is attributable to short maternal stature (31.2% of preterm births in South-Asia and 10.4% of preterm births in sub-Saharan Africa) [24]. Other studies have also concluded that shorter mothers deliver babies prematurely with lower birth weights [24,25]. In our study, and on average, women in Bangladesh were 10.0 cm shorter than women in Zambia. Lower heights could lead to smaller pelvic girdles, which may lead to a higher incidence of fetal growth restriction and obstructed labour [26]. Thus, it would be in the best interest of the fetus to deliver earlier to avoid complications, leading to the argument that shorter gestational age may be an evolutionary adaptation in mothers of some ethnicities [27]. A study suggests anatomic constraints play a more significant role than genetics on premature birth [28].

Our findings on the risk factors of preterm birth corroborate well with previous studies [29,30]. In our study, multiple gestations were the strong risk factor for preterm births. Other risk factors associated with preterm birth included antepartum haemorrhage, pre-eclampsia/ eclampsia, and fever, while clean fuel availability was found to be protective. Three-quarters of children born live before 28 weeks of completed gestation died before completing the first 28 days of life. The risk of mortality decreased subsequently with increasing gestational age, eventually rising slightly in the post-term group (≥42 weeks). This is consistent with previous literature [2].

Our finding highlighted that preterm birth is the key to reduce the neonatal mortality rate further. As many as one in two neonatal deaths occurred in neonates who were born too soon. Furthermore, we observed a high level of mortality in premature infants (14%), many of which could have survived in high-income countries (HIC). In HIC, more than 90% of preterm babies born <28 weeks survive, while in LMICs, they die in the first few days [31]. These findings draw urgent attention to provide adequate and timely intervention, such as antenatal steroid injections for mothers in premature labour, Kangaroo Mother Care (KMC) for preterm /low birth weight babies immediately after birth with respiratory support. A recent study showed that antenatal steroid injection improved neonatal deaths by 16% [32]. Another study showed a 25% reduction in neonatal deaths if KMC was initiated as soon as possible after birth [33]. These simple and inexpensive interventions could save newborn lives.

Assessing accurate gestational age is the first step to identify preterm birth and providing effective measures. In our study, regional differences in the burden of preterm birth disappeared when we used LMP to calculate gestational age. This points towards measurement error of using LMP to estimate gestational age and can be the reason for inconsistencies reported previously in the literature [34]. In our study, we observed a wide limit of agreement between the two measures (-35.2 to 30.1) and that LMP tends to overestimate early gestation and underestimate late gestation, misclassifying term and preterm births in setting where ultrasound access is limited. This implies that preterm newborns may not receive the appropriate level of care if not identified in a timely manner, especially in the early days of life. Therefore, the priority to improve classification and identification of preterm neonates should be to ensure adequate investment in obstetric ultrasound scans in early pregnancy. Concurrently, in a setting where access to ultrasound is limited, we have developed new machine learning models combining newborn characteristics including anthropometrics or LMP, that may predict gestational age within ±15.7-18.4 days of early ultrasound dating. These methods need further testing and could potentially serve as an alternative to early ultrasound dating in low resource settings [35].

Our study highlighted the importance of ensuring women with multiple gestations to be identified early by ultrasound and checked for any potential risk for PROM and other obstetric complications. In addition, comprehensive reproductive history, such as women’s previous history of preterm birth and stillbirths, needs to be examined for recurrent preterm birth. Prevention and diagnosis of maternal morbidity such as pre-eclampsia will help reduce preterm labour. All pregnant adolescents, especially with multiple gestations, should receive appropriate and timely antenatal care, including contraception use and health education activities. Poverty was a risk factor for preterm birth in our study. Lower socioeconomic status, in general, continues to influence developmental delay at two years of age for neonates born early and poor [36]. Programs to focus on early childhood development by improved parenting skills and nurturing the home environment can mitigate the effect of poverty [36].

### Strengths and limitations

There are a few strengths of this study. This study was conducted in five sites in South-Asia and sub-Saharan Africa, which bear the highest burden of adverse birth outcomes. There, high-quality vital information on birth and deaths is sparse or non-existent. Second, this was a large population-based study in which pregnancies are identified by household surveillance of women of reproductive age. The sites also had a birth notification system to ensure early detection and identification of birth outcomes, resulting in low loss to follow up. Third, we used rigorous training to standardize sonographers to performs first-trimester ultrasound dating in all sites. Lastly, we were also able to compare two different methods for calculating gestational age, ie, LMP and ultrasound.

We had a few limitations. First, anthropometric measurements, including the height and weight of the mothers, were not available for all women, which would have been helpful to examine the association of maternal BMI and preterm births. In Pakistan site, in particular, height measurements were available for only one-third of the women. Second, although this was a large population-based study with higher precision on the results, these results may not fully represent the entire country. All study sites are predominantly rural areas except Pakistan (Karachi) site that is peri-urban. Third, we enrolled women presenting at the antenatal care clinic before 20 weeks of gestation. This could lead to a selection bias if women seek antenatal care later in pregnancy have a higher or lower risk of preterm birth, however by study design, we used early ultrasound dating to have accurate gestational age dating.

## CONCLUSIONS

A population-based cohort study showed that the incidence of preterm birth is much higher in South-Asian sites than in sub-Saharan African sites. High rates of neonatal mortality among preterm births, particularly in very and moderate preterm babies, calls for urgent attention to developing policies and intervention packages to improve care around birth and early identification of high-risk pregnancies.

## Additional material


Online Supplementary Document
