# Supplementary material for: Population-based rates, risk factors and consequences of preterm births in South-Asia and sub-Saharan Africa: A multi-country prospective cohort study
Source: J Glob Health. 2022 Feb 19;12:04011. doi: 10.7189/jogh.12.04011 (PMC8850944; doi:10.7189/jogh.12.04011)
Supplement: Online Supplementary Document [file jogh-12-04011-s001.pdf]

Table S1. Risk factors associated with preterm births

	Non-preterm (≥ 37 wks) n=8993	Preterm (<37 wks) n=981	OR* (95% CI)	Adjusted OR (95% CI)
**Maternal and household characteristics[¥]**				
**Pregnant woman's age**				
15-19 years	1043 (89.3)	125 (10.7)	1.19 (0.97, 1.48)	1.27 (1.03, 1.58)
20-34	6041 (90.6)	624 (9.4)	1.0 (reference)	1.0 (reference)
35+	945 (92.8)	74 (7.3)	0.96 (0.74, 1.24)	0.89 (0.69, 1.17)
**Pregnant woman's education**				
No education	1877 (87.6)	265 (12.4)	1.0 (reference)	1.0 (reference)
1-6 years	2818 (91.2)	273 (8.8)	0.97 (0.70, 1.19)	1.02 (0.81, 1.27)
7-12 years	3724 (91.5)	345 (8.5)	0.86 (0.71, 1.06)	0.99 (0.79, 1.25)
13+ years	99 (94.3)	6 (5.7)	0.63 (0.27,1.47)	0.97 (0.42, 2.32)
**Wealth quintile**				
Poorest	1617 (89.6%)	187 (10.4%)	1.39 (1.10, 1.73)	1.36 (1.05, 1.77)
Poorer	1661 (89.6%)	193 (10.4%)	1.39 (1.10, 1.74)	1.36 (1.06, 1.75)
Middle	1645 (89.8%)	186 (10.2%)	1.33 (1.06, 1.67)	1.28 (1.00, 1.64)
Richer	1649 (90.8%)	168 (9.3%)	1.19 (0.94, 1.50)	1.16 (0.92, 1.49)
Richest	1702 (92.1%)	146 (7.9%)	1.0 (reference)	1.0 (reference)
**Previous obstetric history[β]**				
**Previous stillbirth**				
No	6065 (91)	597 (9)	1.0 (reference)	1.0 (reference)
Yes	581 (84.5)	107 (15.6)	1.87 (1.49, 2.35)	1.84 (1.42, 2.37)
**Previous preterm birth**				
No	6443 (90.5)	674 (9.5)	1.0 (reference)	1.0 (reference)
Yes	125 (82.8)	26 (17.2)	1.93 (1.24, 3.00)	1.96 (1.18, 3.26)
**Morbidity during current pregnancy[€]**				
**Antepartum haemorrhage**				
No	7496 (91.0)	743 (9.0)	1.0 (reference)	1.0 (reference)
Yes	113 (87.6)	16 (12.4)	1.23 (0.72, 2.13)	1.24 (0.66, 2.33)
**Pre-eclampsia or eclampsia**				
No	7475 (91.0)	739 (9.0)	1.0 (reference)	1.0 (reference)
Yes	127 (87.0)	19 (13.01)	2.36 (1.42, 3.91)	2.74 (1.56, 4.84)
**Fever before or during delivery**				
No	6504 (91.7)	587 (8.3)	1.0 (reference)	1.0 (reference)
Yes	225 (83)	46 (17)	1.65 (1.12, 2.24)	1.76 (1.18, 2.62)
**Multiple birth**				
Singleton	8861 (91.1)	867 (8.9)	1.0 (reference)	1.0 (reference)
Multiple birth	132 (53.7)	114 (46.3)	13.3 (10.0, 17.8)	14.3 (9.92, 20.52)

* The estimate was adjusted for site.

β The estimates were adjusted for the other factors on previous obstetric history, maternal age, maternal education, wealth quintile, and site.

€ Each maternal morbidity-outcome was analyzed in a separate logistic regression model. Each model was adjusted for maternal age, maternal education, wealth quintile, previous obstetric history and site.

Table S2. Risk factors associated with spontaneous preterm birth

	Non-preterm (≥ 37 wks) n=7863	Preterm (<37 wks) n=761	OR* (95% CI)	Adjusted OR (95% CI)
**Maternal and household characteristics¥**				
**1. Pregnant woman's age**				
15-19 years	916 (89.5)	107 (10.5)	1.33 (1.06, 1.67)	1.41 (1.12, 1.78)
20-34	5369 (91.6)	490 (8.4)	1.0 (reference)	1.0 (reference)
35+	858 (93.3)	62 (6.7)	1.01 (0.76, 1.34)	0.94 (0.70, 1.26)
**2. Pregnant woman's education**				
No education	1729 (88.7)	220 (11.3)	1.0 (reference)	1.0 (reference)
1-6 years	2537 (91.8)	228 (8.3)	0.95 (0.75, 1.19)	1.02(0.80, 1.30)
7-12 years	3211 (92.7)	254 (7.3)	0.81 (0.65, 1.02)	0.98(0.75, 1.26)
13+ years	74 (93.7)	5 (6.3)	0.81 (0.32,2.05)	1.39(0.53, 3.62)
**3. Wealth quintile**				
Poorest	1519 (90.3%)	164 (9.7%)	1.55 (1.19, 2.03)	1.52(1.13, 2.04)
Poorer	1529 (90.1%)	168 (9.9%)	1.59 (1.22, 2.07)	1.55(1.16, 2.06)
Middle	1468 (90.8%)	149 (9.2%)	1.46 (1.11, 1.91)	1.39(1.04, 1.85)
Richer	1402 (91.9%)	124 (8.1%)	1.27 (0.96, 1.68)	1.26(0.95, 1.69)
Richest	1376 (93.6%)	94 (6.4%)	1.0 (reference)	1.0 (reference)
**Previous obstetric historyβ**				
**1. Previous stillbirth**				
No	5497 (91.9)	483 (8.1)	1.0 (reference)	1.0 (reference)
Yes	506 (86.1)	82 (14.0)	1.81 (1.40, 2.34)	1.73(1.30, 2.30)
**2. Previous preterm birth**				
No	5824 (91.5)	541 (8.5)	1.0 (reference)	1.0 (reference)
Yes	108 (84.4)	20 (15.6)	2.00 (1.22, 3.28)	2.00(1.12, 3.55)
**Morbidity during current pregnancy€**				
**1. Antepartum haemorrhage**				
No	6692 (91.7)	603 (8.3)	1.0 (reference)	1.0 (reference)
Yes	101 (87.8)	14 (12.2)	1.35 (0.75, 2.40)	1.42(0.73, 2.76)
**2. Pre-eclampsia or eclampsia**				
No	6674 (91.7)	605 (8.3)	1.0 (reference)	1.0 (reference)
Yes	112 (89.6)	13 (10.4)	1.91 (1.05, 3.48)	2.38(1.25, 4.52)
**3. Fever before or during delivery**				
No	5956 (92.3)	498 (7.7)	1.0 (reference)	1.0 (reference)
Yes	200 (83.0)	41 (17.0)	1.90 (1.31, 2.75)	2.11(1.39, 3.20)
**4. Multiple birth**				
Singleton	7757 (91.9)	681 (8.1)	1.0 (reference)	1.0 (reference)
Multiple birth	106 (57.0)	80 (43.0)	12.38 (8.92, 17.19)	13.42(9.03, 19.95)

* The estimate was adjusted for site.

¥ The estimates were adjusted for all other maternal and household characteristics and site.

β The estimates were adjusted for the other factors on previous obstetric history, six maternal and household characteristics, and site.

€ Each maternal morbidity-outcome was analyzed in a separate logistic regression model. Each model was adjusted for maternal and household characteristics, previous obstetric history and site.
